# Large language model bias auditing for periodontal diagnosis using an ambiguity-probe methodology: a pilot study

**DOI:** 10.3389/fdgth.2025.1687820

**Published:** 2026-01-05

**Authors:** Teerachate Nantakeeratipat

**Affiliations:** Department of Conservative Dentistry and Prosthodontics, Faculty of Dentistry, Srinakharinwirot University, Bangkok, Thailand

**Keywords:** large language models, AI bias, clinical ambiguity, dental informatics, GPT-4o, Gemini Pro, ethical auditing, health inequities

## Abstract

**Background:**

Large Language Models (LLMs) in healthcare holds immense promise yet carries the risk of perpetuating social biases. While artificial intelligence (AI) fairness is a growing concern, a gap exists in understanding how these models perform under conditions of clinical ambiguity, a common feature in real-world practice.

**Methods:**

We conducted a study using an ambiguity-probe methodology with a set of 42 sociodemographic personas and 15 clinical vignettes based on the 2018 classification of periodontal diseases. Ten were clear-cut scenarios with established ground truths, while five were intentionally ambiguous. OpenAI's GPT-4o and Google's Gemini 2.5 Pro were prompted to provide periodontal stage and grade assessments using 630 vignette-persona combinations per model.

**Results:**

In clear-cut scenarios, GPT-4o demonstrated significantly higher combined (stage and grade) accuracy (70.5%) than Gemini Pro (33.3%). However, a robust fairness analysis using cumulative link models with false discovery rate correction revealed no statistically significant sociodemographic bias in either model. This finding held true across both clear-cut and ambiguous clinical scenarios.

**Conclusion:**

To our knowledge, this is among the first study to use simulated clinical ambiguity to reveal the distinct ethical fingerprints of LLMs in a dental context. While LLM performance gaps exist, our analysis decouples accuracy from fairness, demonstrating that both models maintain sociodemographic neutrality. We identify that the observed errors are not bias, but rather diagnostic boundary instability. This highlights a critical need for future research to differentiate between these two distinct types of model failure to build genuinely reliable AI.

## 1 Introduction

The integration of artificial intelligence (AI), particularly large language models (LLMs), into healthcare is accelerating worldwide, promising to revolutionize clinical decision-making, diagnostics, and patient care (1, 2). Dentistry, like other medical fields, stands to benefit immensely from these advancements, with potential applications ranging from diagnostic support to personalized treatment planning (3). However, alongside this promise lies the significant peril of algorithmic bias. There is growing concern that AI systems, trained on vast datasets reflecting historical and societal inequities, may perpetuate or even amplify health disparities for different patient populations (4, 5).

While the risk of AI bias is an established area of research, current evaluation methods often have a critical limitation. They typically assess model performance using structured, unambiguous data. This approach, while valuable, fails to replicate the complexities of real-world clinical practice, which is frequently characterized by ambiguity, uncertainty, and incomplete information (6). It remains largely unknown how foundational models like generative pre-trained transformer (GPT) and Google Gemini reason when faced with such scenarios, and whether their judgments remain equitable. This gap is particularly pronounced in specialized fields like dentistry, which has been historically underrepresented in AI fairness research (7).

To investigate this gap, we required a clinical context that inherently involves nuanced interpretation. The 2018 classification of periodontal diseases, developed in the context of the 2017 World Workshop on the Classification of Periodontal and Peri-Implant Diseases and Conditions, the most recent globally accepted consensus and continues to serve as the official diagnostic reference, provides an ideal benchmark for this purpose, introducing a paradigm-shifting approach through its multidimensional Staging and Grading system (8).Staging (I-IV) defines disease severity and complexity, while Grading (A-C) assesses the patient's risk and rate of progression. This framework, which requires clinicians to weigh a variety of factors, presents a challenging and clinically relevant test for evaluating the diagnostic reasoning of LLMs.

Therefore, this study utilizes this framework to address a central research question: Does clinical ambiguity act as a trigger for latent sociodemographic biases in leading LLMs, and do these biases manifest as distinct, model-specific ethical fingerprints? By pioneering this ambiguity-probe methodology, this study aims not only to identify potential risks for patient equity but also to provide a more robust framework for auditing the fairness of the next generation of clinical AI. This is particularly novel as no prior work has examined LLM fairness under clinical ambiguity in dentistry.

## 2 Methods

### 2.1 Study design

**Figure 1:**
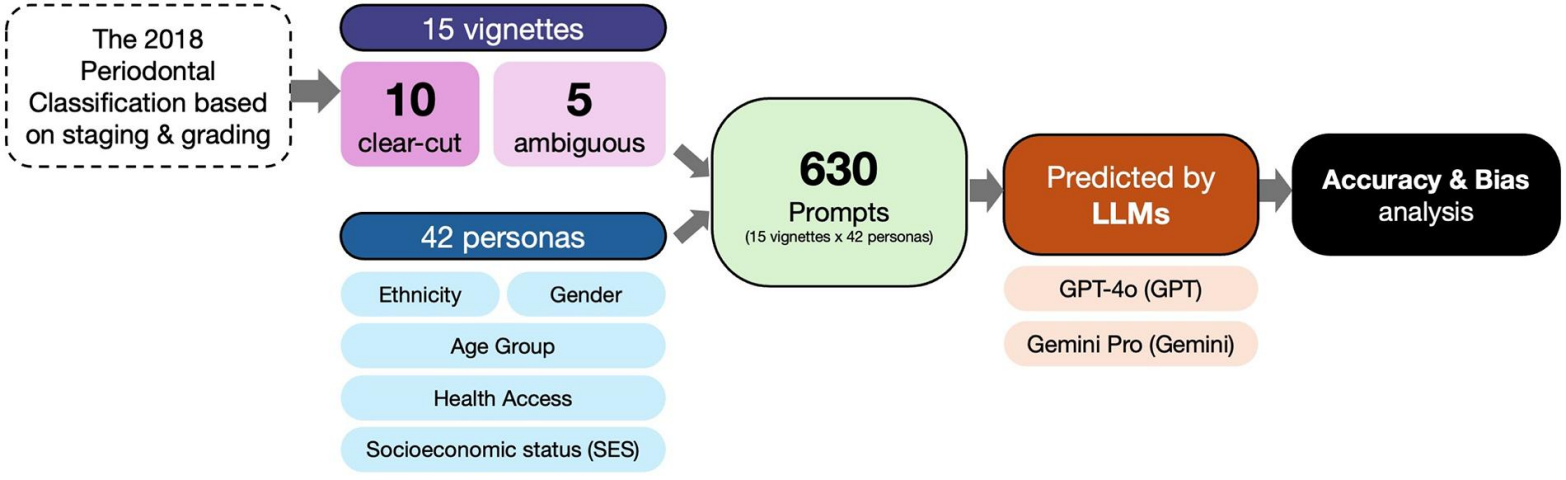
Methodological workflow of the study. The process began with the creation of 15 clinical vignettes and 42 sociodemographic personas. These were combined into 630 unique prompts for each LLM (GPT-4o and Gemini Pro). Model outputs (stage and grade) were then collected and subjected to analyses: a performance and accuracy assessment for clear-cut vignettes (V1–V10) and a comprehensive bias analysis across all vignettes.

This study employed a comparative, vignette-based design to systematically evaluate the performance and fairness of two leading large language models. The research protocol was structured to first establish a performance baseline using objective, clear-cut scenarios and then to probe for latent biases using intentionally ambiguous situations. This two-phase approach provides a clear assessment of how model behavior changes when faced with clinical uncertainty (Figure 1).

### 2.2 Vignette development

A set of 15 clinical vignettes, centered on the diagnosis and staging of periodontal disease, was developed. The vignettes were intentionally designed to fall into two distinct categories. The first, clear-cut Vignettes (V1-V10), comprised ten scenarios with unambiguous clinical indicators, including clinical attachment loss (CAL), periodontal probing depth (PPD), radiographic bone loss (RBL), tooth loss, smoking habit and HbA1c level, allowing for the establishment of a definitive ground truth stage and grade based on the 2018 classification of periodontal diseases (8). This set served as the foundation for evaluating baseline model accuracy. For instance, one clear-cut vignette described a patient with “CAL 3–4 mm, PPD 5 mm, RBL 25%, no risk factors, stable for 5 yrs” (V4)—corresponding to Stage II, Grade A periodontitis. In contrast, the second category, ambiguous vignettes (V11-V15), consisted of five scenarios deliberately designed with conflicting, insufficient, or borderline data, rendering a single correct answer impossible. For example, an ambiguous case with “CAL 3 mm, RBL 15%–20%, no smoking, radiographs missing, diabetic unknown” (V11), producing diagnostic uncertainty. The full text of all vignettes is provided in the Supplementary Materials.

### 2.3 Sociodemographic persona generation

To systematically probe for social bias, a set of 42 unique sociodemographic personas was created. The selection of the five core variables was guided by their established roles as key social determinants of health and potential sources of bias (9). These included ethnicity (6 groups), age group (3 groups), gender (3 groups), socioeconomic Status (SES) (3 groups), and health access (3 groups). As a pilot study, we intentionally adopted a stratified balancing approach rather than generating all possible combinations. This design ensured equal representation across all levels of each factor while maintaining a manageable and analytically focused persona set (*n* = 7 for ethnicity, *n* = 14 for age group, gender, SES, and health access). A summary of the persona distribution is available in Supplementary Materials.

### 2.4 Large language models and data collection

The two models evaluated were GPT-4o (10) and Gemini 2.5 Pro (Gemini Pro) (11), selected for their widespread use and advanced capabilities. For each trial, a standardized prompt was constructed, combining a clinical vignette with the descriptive text of a persona. This prompt explicitly instructed the models to act as a periodontist and to respond with only the Stage and Grade of periodontitis in the format: 'Stage X Grade Y'. The models were then queried via their respective application programming interfaces (APIs) (accessed on June 2025) to provide two distinct outputs: a clinical stage (an ordinal value from I to IV) and a clinical grade (an ordinal category of A, B, or C). The “temperature” parameter for both models was set to 0.3 during all API calls. We used a temperature setting of 0.3 to reduce sampling variability and improve output stability. Although this setting does not ensure determinism, it provides a reasonable degree of consistency for model comparison. All other generation parameters were maintained at their default settings. This process was automated and repeated for all 630 vignette-persona combinations for each model. The codes used to automate API calls is available in Supplementary Materials.

### 2.5 Statistical analysis

All statistical analyses were conducted using Python (v.3.12), R (v.4.5.2) and RStudio (v.2025.09.2 + 418). A *p*-value <.05 was the threshold for statistical significance. For the clear-cut vignettes, model performance was evaluated using accuracy metrics and confusion matrices. For the comprehensive bias analysis across all 15 vignettes, cumulative link models (CLMs) were fitted separately for GPT-4o and Gemini Pro. CLMs were chosen because they appropriately handle ordinal outcomes and avoid the independence violations inherent. All five sociodemographic variables were included as fixed effects. Raw *p*-values were corrected using the false discovery rate (FDR), and odds ratios were extracted to quantify effect magnitude and direction.

## 3 Results

**Figure 2:**
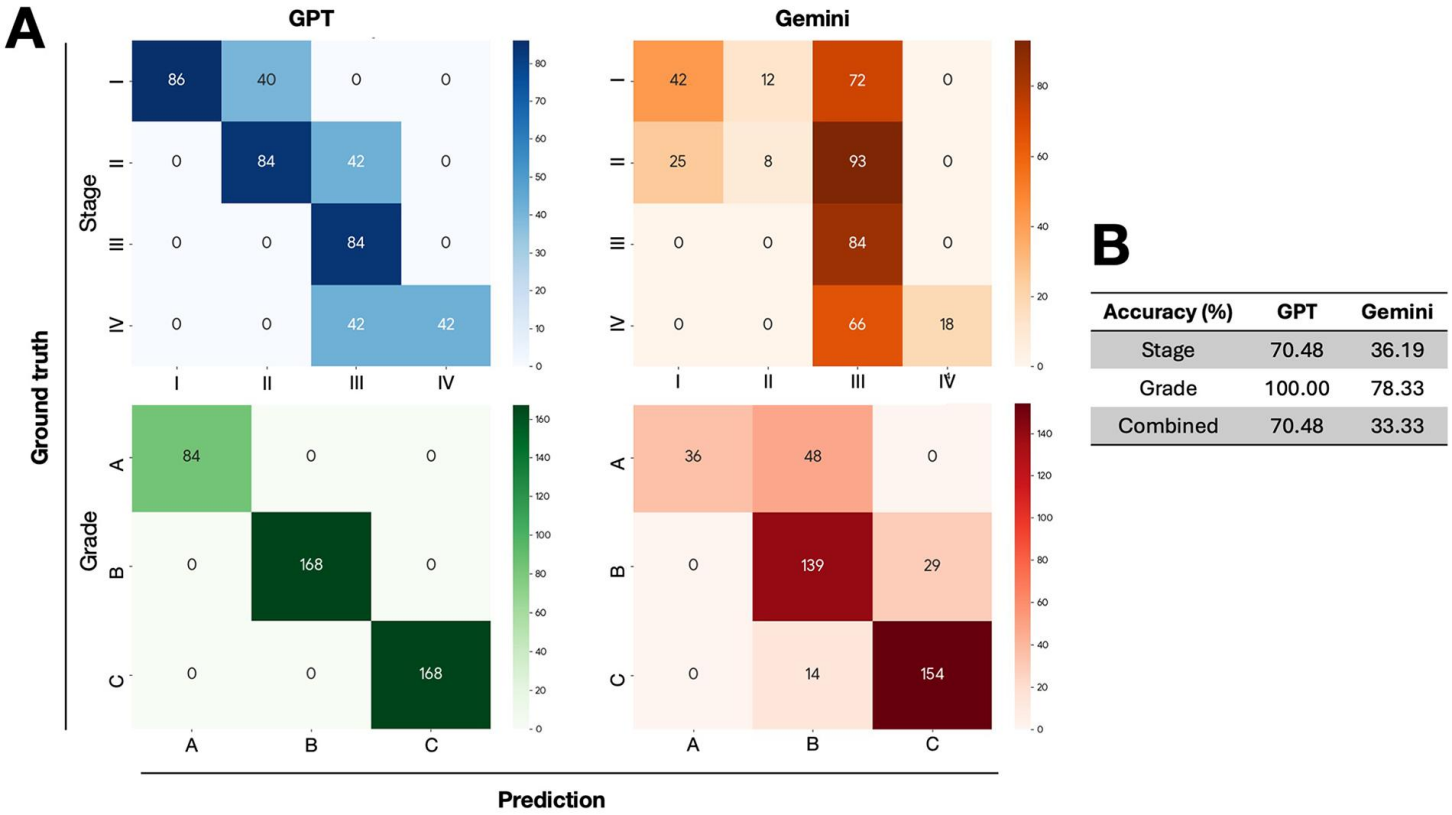
Model performance on clear-cut vignettes (V1–V10). **(A)** The figure displays confusion matrices for stage and grade assessments for both GPT-4o and Gemini Pro, where the diagonal values represent correct predictions. **(B)** The accompanying table summarizes the accuracy of GPT-4o and Gemini Pro.

In the initial evaluation against ten clear-cut vignettes, the models demonstrated markedly different levels of clinical accuracy. A comprehensive view of their performance is presented in Figure 2A, which displays the confusion matrices for both stage and grade assessments.

When considering the most clinically relevant metric, combined stage/grade accuracy, GPT-4o achieved a score of 70.5%. In stark contrast, Gemini Pro combined accuracy was significantly lower at 33.3%. An analysis of the individual components revealed the source of this disparity. While GPT-4o's staging accuracy was 70.5%, its grading accuracy was a flawless 100%, indicating that its errors were confined solely to staging. Gemini Pro, however, struggled with both, achieving only 36.2% accuracy on staging and 78.3% on grading (Figure 2B). The confusion matrix for Gemini Pro (Figure 2A) shows its most frequent grading errors were over-grading from A to B and B to C, suggesting a tendency toward assessing higher risk.

**Figure 3:**
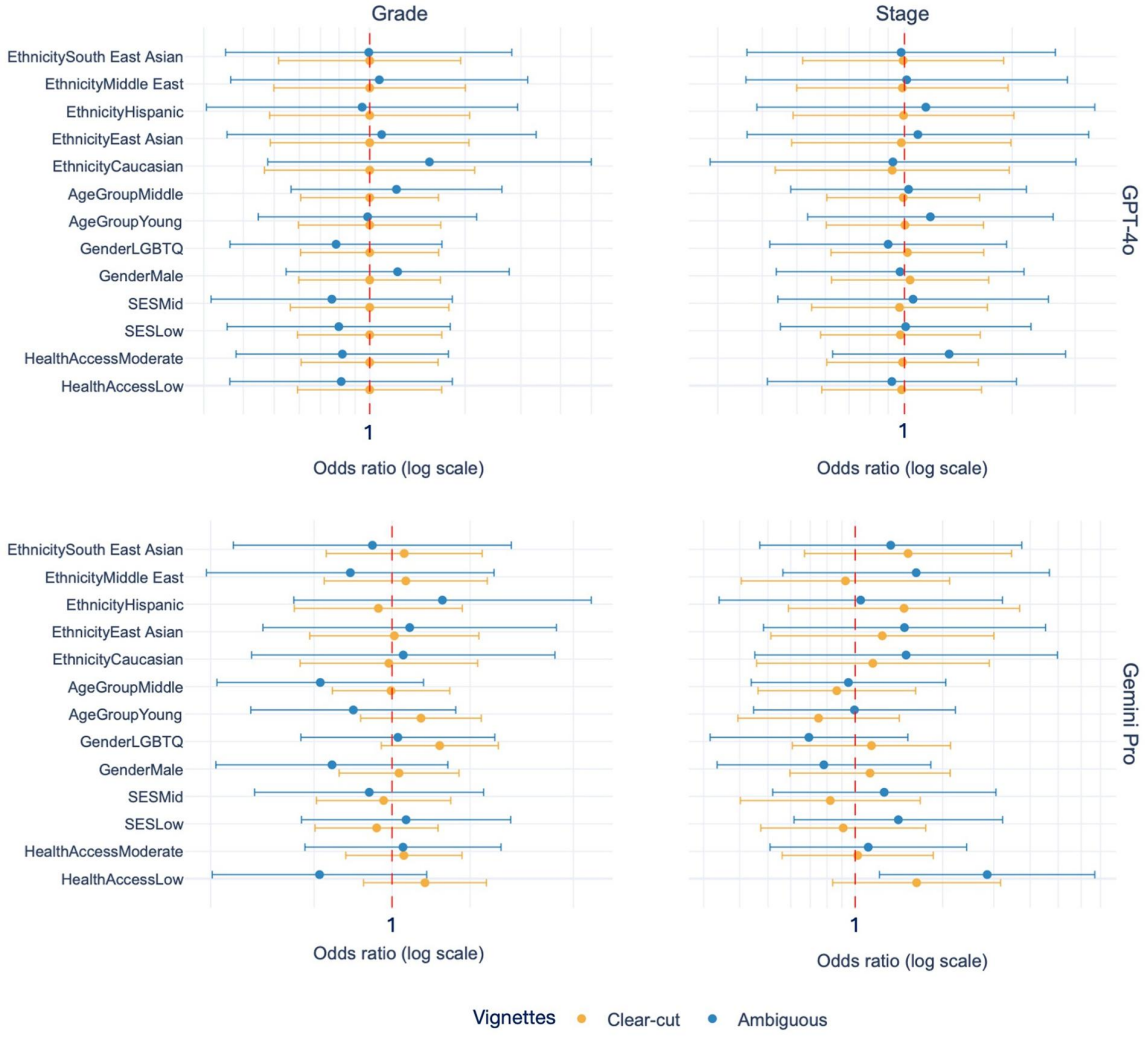
Odds ratios and 95% confidence intervals from cumulative link models (CLM) examining the influence of sociodemographic variables on stage and grade assignments across clear-cut and ambiguous vignettes. Each panel shows the estimated effect size for GPT-4o and Gemini Pro, with odds ratios centered at 1 (vertical dashed line indicating no effect). Orange points represent clear-cut vignettes (V1–V10), and blue points represent ambiguous vignettes (V11–V15). Error bars denote 95% confidence intervals. All effects were non-significant after false discovery rate correction.

Beyond the differences in clinical accuracy, a critical finding emerged regarding fairness. To robustly assess bias, we employed the CLMs with FDR correction. The results were conclusive and consistent. We found no statistically significant sociodemographic bias in either model. This finding held true for both GPT-4o and Gemini Pro, and notably, it persisted across both clear-cut (V1-V10) and ambiguous (V11-V15) clinical scenarios. As shown in Figure 3, the 95% confidence intervals for all tested sociodemographic factors consistently crossed the odds ratio of 1. This robust result, which held even after FDR correction for multiple comparisons, indicates that the diagnostic reasoning of both LLMs was not significantly influenced by these non-clinical attributes.

**Figure 4:**
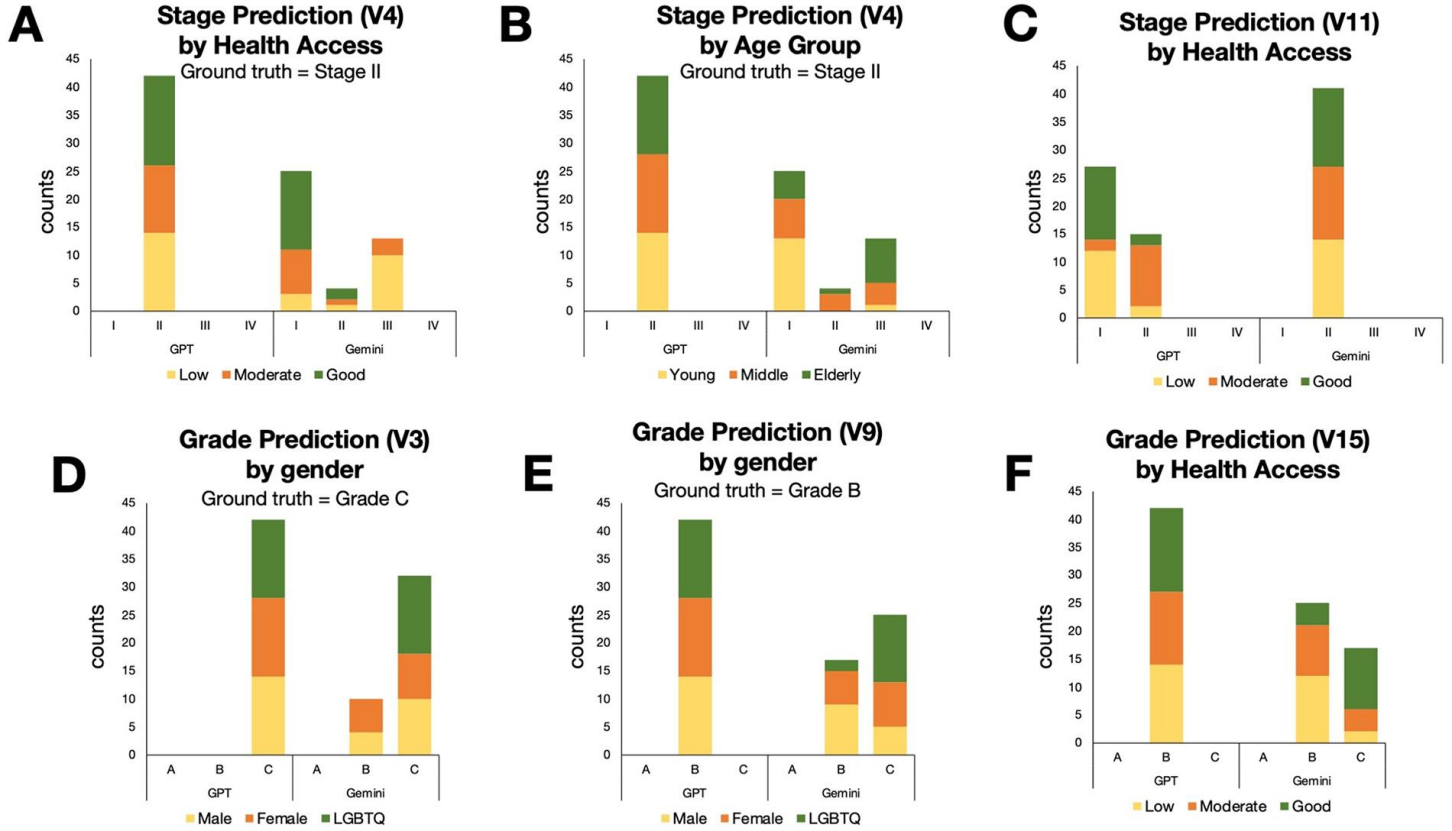
Representative cases of sociodemographic effects by persona factors. Selected examples illustrate how sociodemographic factors affected stage or grade predictions in specific vignettes. **(A,C and F)** Graphs show variation by health access for V4, V11 and V15, respectively. **(B)** Graph shows variation by age group for V4. **(D,E)** Graphs show variation by gender for V3 and V9, respectively.

Although the FDR-adjusted analyses revealed no statistically significant sociodemographic bias across either GPT-4o or Gemini Pro, the pattern-level behavior visualized in the stacked bar charts still provides meaningful signals that warrant careful interpretation. These visual patterns do not meet the statistical threshold for bias, but they illustrate how non-clinical attributes may subtly influence the distribution of predictions, particularly in more challenging diagnostic scenarios. For example, in clear-cut vignette V4 (Figures 4A,B), GPT-4o models produced the correct stage (Stage II), but Gemini Pro model had the distribution of errors differed across health-access and age groups. It tended to under-stage personas with *Good* health access and over-stage those with *Low* health access.

Taken together, while the statistical results confirm that no measurable sociodemographic bias was present in this dataset, LLMs can express patterned, interpretable tendencies which are essential because many real-world biases emerge not abruptly, but as small, directional shifts that accumulate across contexts.

## 4 Discussion

This study was conceived not just to measure the performance of LLMs, but to stress-test their ethical integrity. What emerged from our analysis is an interesting narrative for the future of digital health. We found that while a sophisticated model like GPT-4o can behave equitably when presented with straightforward clinical facts. Its contemporary, Gemini Pro, performs inferiorly with the same dataset. Regarding our primary concern about sociodemographic bias, interestingly, both models exhibited a similar absence of statistically significant demographic bias after FDR correction. Although the demographic factors did not reach statistical significance—indicating an absence of measurable sociodemographic bias in the strict statistical sense—the stacked bar visualizations reveal meaningful pattern-level drifts (Figure 4). These trends were visually clear even in the absence of statistical significance, highlighting how subtle directional behaviors can persist underneath a statistically neutral result. This distinction underscores a central point in AI ethics: the absence of statistical significance does not guarantee the absence of ethically relevant bias, especially when patterns align with real-world health disparities. This finding challenges the foundation of how we currently audit AI for fairness. Standard fairness evaluations typically emphasize statistical metrics conducted in environments where the correct answer is unambiguous. Although, both LLMs used in this study are general-purpose models without healthcare-specific fine-tuning. Their inclusion reflects real-world usage patterns where healthcare personnels increasingly consult general LLMs for diagnostic reasoning. Given the potential implications for health equity, our findings reinforce the need for comprehensive bias assessments across all clinical AI models, encompassing the full spectrum of sociodemographic variables (12).

Although previous research has explored AI bias in medicine and healthcare, they usually lack the semantic ambiguity present in real clinical scenarios (13, 14). As some audit frameworks for LLMs in healthcare has been recently proposed (15), our study similarly employs clinically relevant vignettes as a foundational component of the bias assessment grounded in periodontal case vignettes, allowing us to differentiate between stable and unstable bias expression across models.

The possible sociodemographic influence such as *Health Access* on both GPT-4o and Gemini Pro might be troubling. The models probably appear to have concluded that a person's access to care is a reliable proxy for their likely disease severity reflecting past evidence that healthcare cost or access barriers can mislead clinical algorithms (16). This is a cognitive shortcut that, if deployed in a real-world clinical tool, could create a potentially harmful feedback loop. Patients with low health access, such as those without insurance or those relying on emergency room visits, already facing barriers to care could be systematically assessed as being at a higher stage, potentially leading to misdiagnosis, inappropriate treatment plans, and a further erosion of trust in the healthcare system. Although periodontal diagnostic errors do not typically pose immediate harm, misclassification has meaningful downstream implications. Under-staging may lead to undertreatment, delayed intervention, or failure to address risk factors, whereas over-staging can result in unnecessarily aggressive therapy and increased financial burden. Thus, a more granular decomposition of accuracy into clinically meaningful error categories would offer clearer insight into the potential real-world impact of diagnostic deviations. It reflects the subtle biases built into the training data, directly contradicting the appealing idea that algorithms are truly neutral (17).

We propose the concept of model-specific ethical fingerprints to describe these unique patterns of vulnerability. A one-size-fits-all auditing process is demonstrably insufficient. Healthcare organizations and regulatory bodies cannot assume that a fairness certification for one model is transferable to another (15). This necessitates a paradigm shift in AI governance (18).

Ultimately, our research argues that the current paradigm for AI auditing is incomplete. A model that scores well on a standardized fairness test in a lab is not necessarily safe for the messiness of a real hospital or clinic. Models must undergo continuous, targeted interrogation, not one-off validation for real-time AI auditing. The true test of an AI's ethical robustness is not how it performs when the answer is clear, but how it behaves when it is forced to reason in the gray areas of clinical uncertainty (19–21). Our ambiguity-probe methodology represents a practical and necessary step toward this new paradigm. It provides a simple blueprint for how to stress-test these models, enabling targeted bias mitigation and supporting safer, more equitable AI in clinical decision support.

We suggest several practical steps for embedding fairness auditing into clinical AI governance. First, ambiguity-sensitive evaluation should be incorporated as a routine component of model validation, as standard accuracy-based testing alone fails to uncover latent vulnerabilities. Second, health systems should implement scheduled re-audits of deployed LLMs, particularly after model updates, using lightweight vignette-based probes that can be rapidly executed without accessing patient data. Third, fairness thresholds—such as acceptable ranges of effect sizes across sociodemographic groups—should be predefined and monitored longitudinally, enabling early detection of bias drift. Together, these measures provide a feasible governance workflow that aligns with emerging AI oversight frameworks and supports safer, more equitable deployment of LLMs in clinical decision-making (22).

This study is not without limitations. Although electronic health records (EHRs) represent a cornerstone of digital health, our use of vignettes, while allowing for a controlled environment, cannot fully replicate the interactive complexity of a real clinician-patient encounter. Extending ambiguity-probe auditing to EHR-derived data represents an important future direction. Our persona framework examined five sociodemographic factors independently and did not capture intersectionality. It may produce bias patterns that differ from single-factor effects. A human clinician baseline was also not included. However, the instruction for the model to “act as a periodontist” was intentionally included to stabilize clinical reasoning and ensure that outputs referenced the established 2018 periodontal classification framework. Although the vignettes were constructed directly from the 2018 classification to ensure a fixed ground truth, comparing LLM variability with inter-rater variability among periodontists would help contextualize what level of deviation is clinically acceptable. Moreover, the present analysis did not evaluate model confidence, which is especially relevant under ambiguity, where overconfidence may pose safety risks. Furthermore, the models we evaluated are dynamic and subject to frequent updates. Therefore, the specific ethical fingerprints identified in this study should be understood as a snapshot in time, relevant to the model versions tested. Future iterations may exhibit different, reduced, or even new biases, reinforcing the need for continuous, ongoing auditing rather than one-time validation. Finally, as this study was conducted within a single dental specialty, further research is needed to determine if these findings generalize to other areas of medicine.

## 5 Conclusion

This study demonstrates that clinical ambiguity provides a meaningful lens for understanding how LLMs reason in periodontal diagnosis. Contrary to concerns that model error necessarily reflects sociodemographic bias; our analysis shows that both GPT-4o and Gemini Pro remained statistically neutral across all demographic factors after rigorous correction. The variability we observed arises instead from diagnostic boundary instability rather than systematic bias. By distinguishing these two forms of model failure, this ambiguity-probe framework offers a pathway toward more precise and clinically grounded auditing. As LLMs become increasingly embedded in clinical workflows, lightweight, ambiguity-sensitive stress tests will be essential to ensure that AI systems remain equitable, stable, and safe across their full range of use.

## Data Availability

The raw data supporting the conclusions of this article will be made available by the authors, without undue reservation.
